# Clinical characteristics of new‐onset diabetes after liver transplantation and outcomes

**DOI:** 10.1002/ags3.12775

**Published:** 2024-01-19

**Authors:** Shingo Shimada, Katsunori Miyake, Deepak Venkat, Humberto Gonzalez, Dilip Moonka, Atsushi Yoshida, Marwan Abouljoud, Shunji Nagai

**Affiliations:** ^1^ Division of Transplant and Hepatobiliary Surgery Henry Ford Health System Detroit Michigan USA; ^2^ Division of Gastroenterology and Hepatology Henry Ford Health System Detroit Michigan USA

**Keywords:** immunosuppression, liver transplantation, long‐term outcomes, new‐onset diabetes

## Abstract

**Background:**

We aimed to identify the characteristics of new‐onset diabetes after liver transplantation (LT) (NODAT) and investigate its impacts on post‐transplant outcomes.

**Methods:**

Adult LT patients between 2014 and 2020 who used tacrolimus as initial immunosuppression and survived 3 months at least were evaluated. Patients who developed NODAT within 3 months after LT were classified as NODAT group. Also, patients were further classified as history of diabetes before LT (PHDBT) and non‐diabetes (ND) groups. Patient characteristics, post‐LT outcomes, and cardiovascular and/or pulmonary complications were compared.

**Results:**

A total of 83, 225, and 263 patients were classified into NODAT, PHDBT, and ND groups. The proportion of cholestatic liver disease and rejection within 90 days were higher in NODAT group. Mean serum tacrolimus concentration trough level in the first week after LT was 7.12, 6.12, and 6.12 ng/mL (*p* < 0.001). Duration of corticosteroids was significantly longer in NODAT compared to PHDBD or ND (416, 289, and 228 days, *p* < 0.001). Three‐year graft and patient survival were significantly worse in NODAT than ND (80.5% vs. 95.0%, *p* < 0.001: 82.0% vs. 95.4%, *p* < 0.001) but similar to PHDBT. Adjusted risks of 3‐year graft loss and patient death using Cox regression analysis were significantly higher in NODAT compared to ND (adjusted hazard ratio [aHR] 3.41, *p* = 0.004; aHR 3.61, *p* = 0.004). Incidence rates of cardiovascular or pulmonary complications after LT in NODAT were significantly higher than ND but similar to PHDBT.

**Conclusion:**

Higher initial tacrolimus concentration and early rejection might be risk factors for NODAT. NODAT was associated with worse post‐transplant outcomes.

## Graphical abstract

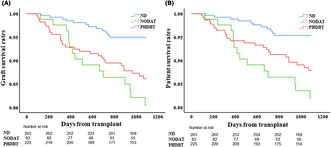

## 1 INTRODUCTION

In the general population, the prevalence of diabetes is estimated to be up to 4%.^1^ Meanwhile, new‐onset diabetes after transplantation (NODAT) is a frequent comorbidity for patients who received solid organ transplantation, which is estimated to be up to 7%–28% in patients after liver transplantation (LT).^2^ Steroids increase insulin resistance and gluconeogenesis,^3^ and calcineurin inhibitors impair insulin secretion from pancreatic βcells.^4^ Therefore, steroids and calcineurin inhibitors were well‐known as risk factors for NODAT.^5^, ^6^ In addition, various risk factors such as older age,^7^ hepatitis C virus (HCV),^8^ and acute cellular rejection (ACR)^9^ were reported as risk factors for NODAT in other studies.

Diabetes is a risk factor for cardiovascular events^10^ and/or infectious diseases.^11^ In patients who received LT, a history of diabetes increases the risk of mortality following LT,^12^ which were often related to atherosclerotic vascular events^13^ and/or end‐stage kidney disease.^14^

Meanwhile, the impact of NODAT on graft loss, mortality, and cardiovascular events were still controversial.^15^, ^16^, ^17^ Kuo et al. reported that a history of diabetes was associated with mortality and graft failure but not NODAT.^15^ According to the large Korean multicenter study, graft survival rates were similar regardless of NODAT.^16^ Other reports showed that patients with NODAT had reduced survival and an increased incidence of sepsis and chronic renal insufficiency.^17^

The aim of this study is to identify risk factors for NODAT, and to investigate the impact of the NODAT on graft and patient survival, and cardiovascular events compared to patients who didn't show diabetes or patients who had diabetes before LT.

## 2 METHODS

### 2.1 Study population

**FIGURE 1:**
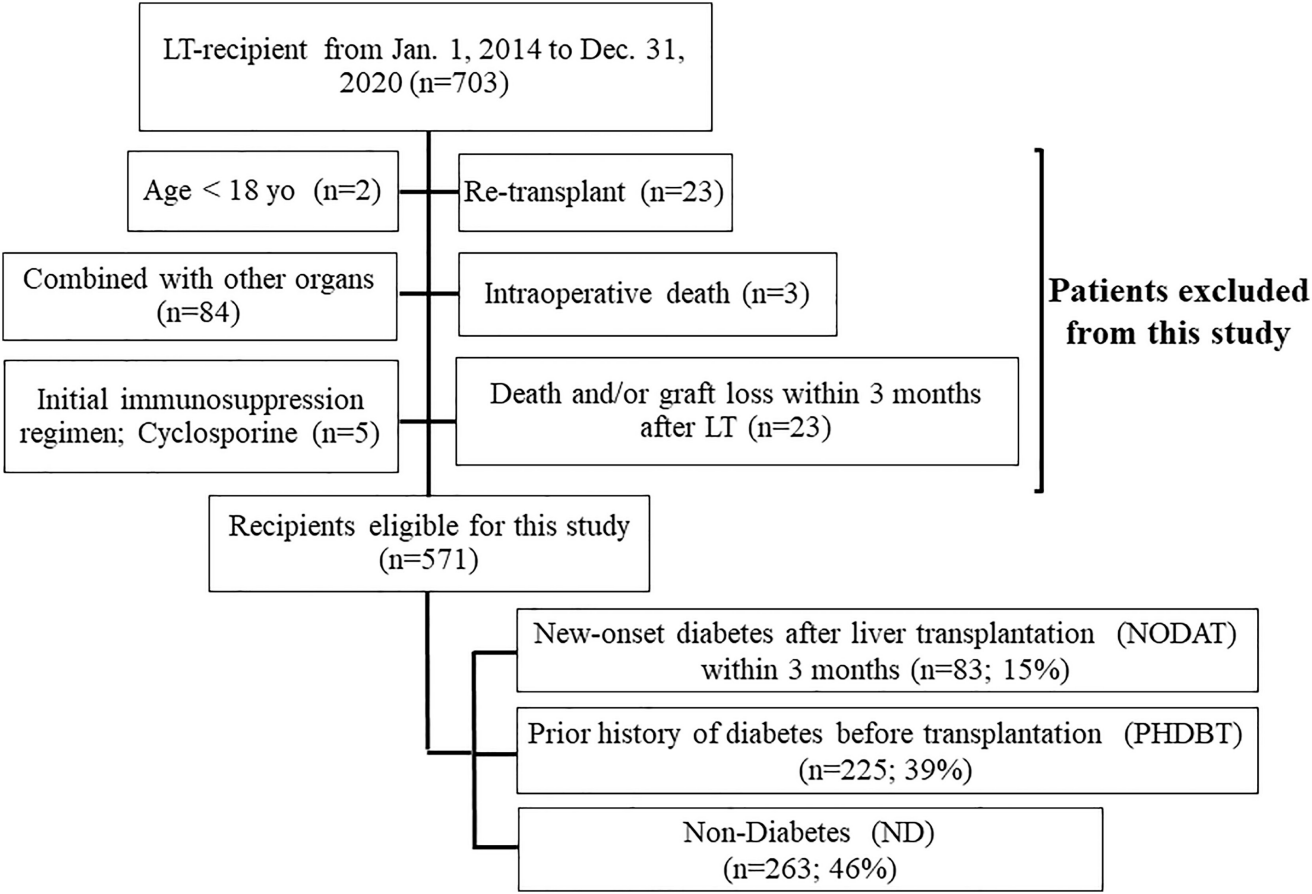
Flow chart of study population selection.

Henry Ford Health (HFH) is an integrated tertiary care center in metropolitan Detroit, Michigan, US. Study protocols were approved by the HFH Institutional Review Board (#15051); requirements for written informed consent were waived due to the de‐identified and observational nature of data. Retrospective medical records data were collected for patients who received a liver transplant (LT) between January 2014 and December 2020. Adult patients (≥18 years) who used tacrolimus as initial immunosuppression and survived 3 months at least were eligible for inclusion. Patients who received retransplant or combined transplants with thoracic organs, intestine, and/or pancreas were excluded. Three patient who experienced intraoperative death was excluded. Patients who developed new‐onset diabetes after LT (NODAT) within 3 months after LT were classified as NODAT group. Also, patients were further classified as previous history of diabetes before LT (PHDBT) and non‐diabetes (ND) groups (Figure 1).

### 2.2 The definition of NODAT

The definition of NODAT is as follows^18^: 1) two posttransplant fasting plasma glucose levels ≥126 mg/dL ≥30 days apart; 2) oral hypoglycemic agent use for ≥30 consecutive days after transplantation; 3) insulin therapy for ≥30 consecutive days after transplantation; 4) hemoglobin A1c (HbA1c) ≥6.5% on at least one occasion after transplantation. Patients who have at least one of four criteria are diagnosed as NODAT.

### 2.3 Covariates

Categorical variables included: recipient/donor gender; recipient/donor race (White, Black, Hispanic, other); etiology of end‐stage liver disease (hepatitis B virus [HBV], hepatitis C virus [HCV], nonalcoholic steatohepatitis [NASH], cholestatic disease, alcohol‐related liver disease); Karnofsky score at LT (10–30%, 40%–60%, or 70%–100%); presence of severe/moderate grade ascites at LT (y/n); grade III/IV encephalopathy at LT (y/n); dialysis at LT (y/n); mechanical ventilation at LT (y/n); presence of hepatocellular carcinoma (HCC) at LT (y/n); recipient family history of diabetes within second degree relative (y/n); type of liver graft (whole or partial/split); use of donation after circulatory death (DCD) donor liver graft (y/n); donor cause of death (trauma, anoxia, cerebrovascular accident [CVA], or other); rejection within 90 days after LT (biopsy proven) (y/n); steroid pulse treatment within 90 days after LT (y/n); reoperation within 30 days after LT (y/n); and readmission within 30 days after LT (y/n); patients who could stop corticosteroids (y/n).

Recipient/donor age at LT, recipient/donor body mass index (BMI) at LT, recipient white blood cell (WBC) at LT, hemoglobin (Hb) at LT, platelet (Plt) at LT, total cholesterol (T‐cho) at LT, HbA1c at LT, albumin (Alb) at LT, recipient model for end‐stage liver disease (MELD) score at LT, recipient warm ischemia time (WIT), recipient cold ischemia time (CIT), amount of blood loss at LT, operative time at LT, amount of intraoperative transfusion (red blood cell [RBC], fresh frozen plasma [FFP], platelet concentrates [PC]), hospital stay days after LT, ICU stay days after LT, mean tacrolimus trough in the first week after LT, duration of corticosteroids after LT were used as continuous variables.

### 2.4 Comparison of patient characteristics

Patient characteristics were compared among the three groups (NODAT vs. PHDBT vs. ND). These were also compared between the two groups (NODAT vs. ND). Multivariable analysis was performed to identify the risk factors for NODAT using logistic regression after univariable analysis.

### 2.5 Comparison of post‐LT outcomes

Three‐year graft and patient survival after LT were compared among the three groups. We performed multivariable analyses of risk factors for post‐LT 3‐year graft loss and patient death. Cardiovascular events, pulmonary complications, and/or the proportion of patients who could stop the medication for diabetes after LT were also compared.

### 2.6 Statistical analysis

Patient and donor demographic and clinical characteristics were described by the groups, using median and interquartile range (IQR) for continuous variables and numbers and percentages for categorical variables. Continuous variables were compared with the Mann–Whitney *U* test and categorical variables were compared using the chi‐square test. Logistic regression was used for the multivariable analysis to identify the risk factors for NODAT. Post‐transplant graft and patient survival were evaluated using the Kaplan–Meier method and groups were compared using log‐rank tests. A multivariable Cox regression model assessed hazards of post‐transplant graft loss and patient death using factors which had *p* value <0.157 in univariable analyses.^19^ *p*‐values <0.05 were considered statistically significant for all analyses. All statistical analyses were completed using SPSS version 27 (IBM, Chicago IL, USA) and R version 3.5.1 (R Foundation for Statistical Computing, Vienna, Austria).

## 3 RESULTS

### 3.1 Patient characteristics

A total of 83, 225, and 263 patients were classified into NODAT, PHDBT, and ND groups (Figure 1). The proportion of patients who had cholestatic liver disease (22, 5, and 13%, *p* < 0.001), rejection within 90 days (54, 15, and 7%, *p* < 0.001), and steroid pulse treatment within 90 days (16, 5, and 2%, *p* < 0.001) were significantly higher in NODAT compared to PHDBT or ND. Mean serum tacrolimus concentration trough level in the first week after LT was higher in NODAT (7.12, 6.12, and 6.12 ng/mL, *p* < 0.001). Duration of corticosteroids was significantly longer (416, 289, and 228 days, *p* < 0.001) in NODAT. Recipient age (61, 58, and 58 yo, *p* = 0.01), the proportion of patients who had NASH (40, 13, and 13%, *p* < 0.001), and family history of diabetes within second‐degree relative (45, 30, and 26%, *p* < 0.001) were significantly higher in PHDBT compared to NODAT or ND. Recipient BMI (29.5, 28.3, and 28.3 kg/m2, *p* < 0.04), HbA1c (6.0, 5.1, and 4.9%, *p* < 0.001), and amount of intraoperative blood loss (2000, 1750, and 1500 mL, *p* = 0.004) were significantly higher in PHDBT (Table 1).

**TABLE 1:** Comparison of recipient and donor characteristics among patients stratified by the diabetes status.

Characteristics	Group	NODAT	PHDBT	ND	*p*
		*N* = 83	*N* = 225	*N* = 263	
Recipient age (year), median [IQR]		58 [50, 63]	61 [54, 64]	58 [51, 64]	**0.01**
Recipient gender, *n* (%)	Male	52 (63)	159 (71)	163 (62)	0.11
	Female	31 (37)	66 (29)	100 (38)	
Recipient BMI (kg/m^2^), median [IQR]		28.3 [25.1, 31.9]	29.5 [26.2, 33.6]	28.3 [24.6, 31.8]	**0.04**
HBV, *n* (%)		2 (2)	6 (3)	1 (0.4)	0.10
HCV, *n* (%)		22 (27)	47 (21)	78 (30)	0.08
NASH, *n* (%)		11 (13)	89 (40)	35 (13)	**<0.001**
Alcohol, *n* (%)		33 (40)	73 (32)	110 (42)	0.09
Cholestatic disease, *n* (%)		18 (22)	12 (5)	34 (13)	**<0.001**
HCC, *n* (%)		21 (25)	58 (26)	63 (24)	0.89
Recipient race, *n* (%)	White	65 (78)	187 (83)	224 (85)	0.22
	Black	14 (17)	18 (8)	25 (10)	
	Hispanic	3 (4)	14 (6)	9 (3)	
	Others	1 (1)	6 (3)	5 (2)	
Dialysis, *n* (%)		2 (2)	10 (4)	8 (3)	0.59
Mechanical ventilation, *n* (%)		1 (1)	4 (2)	4 (2)	0.93
Karnofsky score (%), *n* (%)	70–100	9 (11)	24 (11)	23 (9)	0.59
	40–60	65 (78)	166 (74)	193 (73)	
	10–30	9 (11)	35 (16)	47 (18)	
Severe/Moderate Ascites, *n* (%)		26 (31)	82 (36)	102 (39)	0.46
Grade III/IV encephalopathy, *n* (%)		5 (6)	33 (15)	40 (15)	0.08
Family history of diabetes within second degree relative, *n* (%)		25 (30)	102 (45)	69 (26)	**<0.001**
MELD score, median [IQR]		19 [14, 26]	20 [15, 26]	22 [15, 29]	0.19
Albumin (g/dL), median [IQR]		2.9 [2.5, 3.5]	3.0 [2.6, 3.5]	3.1 [2.7, 3.5]	0.30
WBC (/μL), median [IQR]		5100 [4300, 7100]	4900 [3600, 7200]	5300 [4000, 7200]	0.16
Hb (g/dL), median [IQR]		11.1 [9.1, 12.3]	10.7 [9.0, 12.3]	10.9 [9.0, 12.9]	0.45
Plt (×10^4^/μL), median [IQR]		9.8 [6.9, 13.5]	8.2 [5.8, 11.6]	8.8 [6.1, 12.7]	0.13
T‐cho (mg/dL), median [IQR]		131 [95, 164]	117 [84, 147]	120 [74, 153]	0.08
HbA1c (%), median [IQR]		5.1 [4.6, 5.5]	6.0 [5.4, 7.2]	4.9 [4.4, 5.3]	**<0.001**
Operative time at LT (min), median [IQR]		391 [355, 461]	389 [332, 464]	385 [326, 442]	0.16
Amount of blood loss at LT (mL), median [IQR]		1750 [1000, 4275]	2000 [1175, 3500]	1500 [975, 2500]	**0.004**
Intraoperative RBC at LT (units), median [IQR]		3 [0, 6]	3 [1, 7]	3 [1, 5]	**0.02**
Intraoperative FFP at LT (units), median [IQR]		4 [1, 9]	6 [2, 10]	5 [2, 8]	0.16
Intraoperative PC at LT (units), median [IQR]		0 [0, 1]	0 [0, 1]	0 [0, 1]	0.16
Recipient warm ischemia time (min), median [IQR]		37 [29, 46]	36 [30, 42]	35 [29, 41]	0.32
Graft type, *n* (%)	Whole	74 (89)	203 (90)	240 (91)	0.83
	Partial/Split	9 (11)	22 (10)	23 (9)	
Donor age (year), median [IQR]		45 [33, 54]	41 [29, 54]	43 [30, 55]	0.34
Donor gender, *n* (%)	Male	53 (64)	142 (63)	142 (54)	0.07
	Female	30 (36)	83 (37)	121 (46)	
Cold ischemia time (hours), median [IQR]		4.9 [4.0, 5.9]	5.1 [4.2, 6.0]	4.9 [4.1, 5.8]	0.25
Donor race, *n* (%)	White	66 (79)	174 (77)	197 (75)	0.94
	Black	13 (16)	39 (17)	52 (20)	
	Hispanic	4 (5)	11 (5)	12 (4)	
	Others	0 (0)	1 (0.4)	2 (1)	
Donor BMI (kg/m^2^), median [IQR]		27.0 [23.4, 32.0]	28.3 [24.1, 31.8]	28.0 [24.0, 31.9]	0.54
DCD donor, *n* (%)		12 (15)	31 (14)	33 (13)	0.87
Donor cause of death, *n* (%)	Trauma	16 (19)	50 (22)	47 (18)	0.79
	Anoxia	41 (49)	95 (42)	119 (45)	
	CVA	17 (21)	59 (26)	69 (26)	
	Others	9 (11)	21 (9)	28 (11)	
Hospital stay after LT (days), median [IQR]		10 [7, 15]	9 [7, 14]	9 [7, 12]	0.06
ICU stay after LT (days), median [IQR]		3 [2, 4]	3 [2, 4]	3 [2, 4]	0.30
Tacrolimus trough in the first week after LT (ng/mL), median [IQR]		7.12 [5.87, 8.93]	6.12 [4.68, 7.47]	6.12 [4.81, 7.39]	**<0.001**
Duration of corticosteroids after LT (days), median [IQR]		416 [208, 1084]	289 [124, 720]	228 [119, 538]	**<0.001**
Patients who could stop corticosteroids, *n* (%)		59 (71)	199 (88)	236 (90)	**<0.001**
Rejection within 90 days after LT, *n* (%)		45 (54)	33 (15)	19 (7)	**<0.001**
Steroid pulse treatment within 90 days after LT, *n* (%)		13 (16)	11 (5)	6 (2)	**<0.001**
Reoperation within 30 days after LT, *n* (%)		9 (11)	22 (10)	29 (11)	0.89
Readmission within 30 days after LT, *n* (%)		22 (27)	44 (20)	47 (18)	0.22

*Note*: Data was summarized using the median with interquartile range (IQR) for continuous variables and using percentage for discrete variables. Continuous variables were analyzed using the Mann–Whitney *U* test and discrete variables were analyzed using a chi‐square test.

Abbreviations: BMI, body mass index; CVA, cerebrovascular accident; DCD, donation after circulatory death; FFP, fresh frozen plasma; Hb, hemoglobin; HbA1c, hemoglobin A1c; HBV, hepatitis B virus; HCC, hepatocellular carcinoma; HCV, hepatitis C virus; ICU, intensive care unit; LT, liver transplant; MELD, model for end‐stage liver disease; NASH, non‐alcoholic steatohepatitis; ND, non‐diabetes; NODAT, new‐onset diabetes after transplantation; PC, platelet concentrates; PHDBT, prior history of diabetes before transplantation; Plt, platelet; RBC, red blood cell; T‐cho, total cholesterol; WBC, white blood cell.

### 3.2 Risk factors for NODAT

Compared to ND, HbA1c (5.1 vs. 4.9%, *p* = 0.01), total cholesterol (131 vs. 120 mg/dL, *p* = 0.03), the proportion of patients who had grade 3 or 4 encephalopathy (6 vs. 15%, *p* = 0.04), rejection within 90 days (54 vs. 7%, *p* < 0.001), and steroid pulse treatment within 90 days (16 vs. 2%, *p* < 0.001), mean serum tacrolimus concentration trough level in the first week after LT (7.12, vs. 6.12%, *p* < 0.001) were significantly higher in NODAT by univariable analyses.

Multivariable analysis showed that mean serum tacrolimus concentration trough level in the first week after LT (odds ratio [OR] 1.11, 95% confidence interval [CI] 1.01–1.23, *p* = 0.03) and the proportion of patients who had rejection within 90 days (OR 6.10, 95% CI 3.06–12.10, *p* < 0.001), and HbA1c (OR 4.65, 95% CI 3.41–6.34, *p* < 0.001) were significant risk factors for NODAT (Table 2).

**TABLE 2:** The impact of patient characteristics for NODAT.

	OR	95% CI	*p*
Rejection within 90 days after LT	6.10	3.06–12.10	**<0.001**
HbA1c	4.65	3.41–6.34	**<0.001**
Tacrolimus trough in the first week after LT	1.11	1.01–1.23	**0.03**
Steroid pulse treatment within 90 days after LT	1.23	0.39–3.83	0.72
Total cholesterol	1.00	0.99–1.00	0.08
Grade III/IV encephalopathy	0.89	0.49–1.59	0.68

Abbreviations: CI, confidence interval; HbA1c, hemoglobin A1c; LT, liver transplant; NODAT, new‐onset diabetes after transplantation; OR, odds ratio.

### 3.3 The impact of NODAT on post‐LT outcomes

**FIGURE 2:**
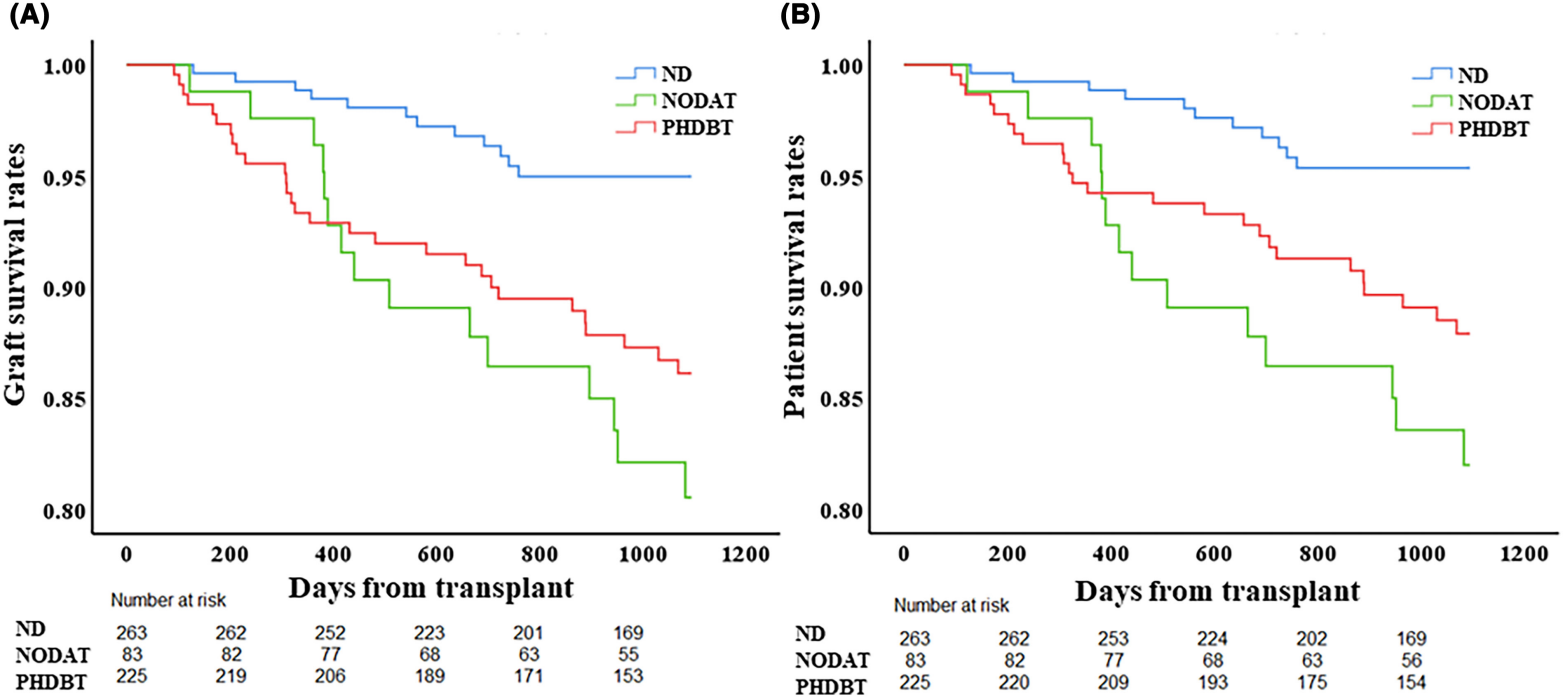
Comparison of post‐LT outcome among the three groups stratified by the diabetes status. (A) Three‐year graft survival rate in NODAT was significantly lower than in ND (80.5% vs. 95.0%, *p* < 0.001) but similar to those in PHDBT (80.5% vs. 86.1%, *p* = 0.30). (B) Three‐year patient survival rate in NODAT was significantly lower than in ND (82.0% vs. 95.4%, *p* < 0.001) but similar to those in PHDBT (82.0% vs. 87.9%, *p* = 0.20).

Three‐year graft and patient survival rates in NODAT were significantly lower than those in ND (graft and patient: 80.5% vs. 95.0%, *p* < 0.001, 82.0% vs. 95.4%, *p* < 0.001) but similar to PHDBT (*p* = 0.30, *p* = 0.20; Figure 2A,B). Multivariable analysis showed the following covariates were associated with significantly increased risk of graft loss: NODAT (ref. ND; adjusted hazard ratio [aHR] 3.41, 95% CI 1.47–7.89, *p* = 0.004); PHDBT (ref. ND; aHR 3.24, 95% CI 1.60–6.54, *p* = 0.001); duration of corticosteroids (aHR 1.01, 95% CI 1.00–1.01, <0.001); hospital stay (aHR 1.05, 95% CI 1.02–1.08, *p* < 0.001); presence of HCC (aHR 2.48, 95% CI 1.38–4.48, *p* = 0.002), patients who could not stop corticosteroids (aHR 14.29, 95% CI 7.14–33.33, *p* < 0.001) (Table 3). NODAT (ref. ND; aHR 3.61, 95% CI 1.50–8.66, *p* = 0.004), PHDBT (ref. ND; aHR 3.21, 95% CI 1.52–6.79, *p* = 0.002), duration of corticosteroids (aHR 1.01, 95% CI 1.00–1.01, <0.001), hospital stay (aHR 1.04, 95% CI 1.02–1.07, *p* < 0.001), presence of HCC (aHR 3.64, 95% CI 1.88–7.04, *p* < 0.001), patients who could not stop corticosteroids (aHR 20.00, 95% CI 9.09–50.00, *p* < 0.001), and MELD score (aHR 1.05, 95% CI 1.01–1.09, *p* = 0.008) were also shown as significant covariates in patient death (Table 3).

**TABLE 3:** Risks for 3‐year graft loss and patient death after liver transplantation.

Factors	aHR	95% CI	*p* Value
Graft loss			
NODAT [ref. ND]	3.41	1.47–7.89	**<0.001**
PHDBT [ref. ND]	3.24	1.60–6.54	**<0.001**
Duration of corticosteroids after LT	1.01	1.00–1.01	**<0.001**
Patients who could stop corticosteroids	0.07	0.03–0.14	**<0.001**
Hospital stay after LT	1.05	1.02–1.08	**<0.001**
HCC	2.48	1.38–4.48	**0.002**
Donor cause of death Anoxia [ref. Trauma]	1.21	0.60–2.45	0.60
Donor cause of death CVA [ref. Trauma]	0.45	0.15–1.35	0.15
Donor cause of death Others [ref. Trauma]	1.12	0.41–3.05	0.82
Recipient race Black [ref. White]	1.82	0.88–3.78	0.11
Recipient race Hispanic [ref. White]	0.48	0.06–3.55	0.47
Karnofsky score 10%–30% [ref. 70%–100%]	4.63	0.56–38.18	0.15
Karnofsky score 40%–60% [ref. 70%–100%]	4.33	0.58–32.00	0.15
Tacrolimus trough in the first week after LT	1.09	0.97–1.22	0.17
ICU stay after LT	0.99	0.94–1.05	0.75
Patient death			
NODAT [ref. ND]	3.61	1.50–8.66	**0.004**
PHDBT [ref. ND]	3.21	1.52–6.79	**0.002**
Duration of corticosteroids after LT	1.01	1.00–1.01	**<0.001**
Patients who could stop corticosteroids	0.05	0.02–0.11	**<0.001**
Hospital stay after LT	1.04	1.02–1.07	**<0.001**
HCC	3.64	1.88–7.04	**<0.001**
MELD score at LT	1.05	1.01–1.09	**0.008**
Recipient race Black [ref. White]	2.16	0.98–4.77	0.056
Karnofsky score 10%–30% [ref. 70%–100%]	4.63	0.56–38.18	0.23
Karnofsky score 40%–60% [ref. 70%–100%]	4.33	0.58–32.00	0.16
Tacrolimus trough in the first week after LT	1.10	0.97–1.25	0.14
Recipient gender Male	1.69	0.79–3.57	0.18

Abbreviations: aHR, adjusted hazard ratio; CI, confidence interval; CVA, cerebrovascular accident; HCC, hepatocellular carcinoma; ICU, intensive care unit; LT, liver transplant; MELD, model for end‐stage liver disease; ND, non‐diabetes; NODAT, new‐onset diabetes after transplantation; PHDBT, prior history of diabetes before transplantation.

Incidence rates of cardiovascular or pulmonary complications after LT in NODAT were significantly higher than in ND but similar to PHDBT (cardiovascular complication; 16.9%, 3.8%, and 16.9%, *p* < 0.001, *p* = 1.00, pulmonary complication; 20.5%, 11.0%, and 20.0%, *p* = 0.04, *p* = 1.00) (Table 4). NODAT also had a significantly higher proportion of patients who could stop the medication for diabetes after LT compared to PHDBT (51.8% vs. 6.7%, *p* < 0.001) (Table 5). The cause of 3‐year mortality was shown in Table S1.

**TABLE 4:** Comparison of complications after liver transplantation.

Complications	NODAT	PHDBT	ND	*p*	*p*
	*N* = 83	*N* = 225	*N* = 263	NODAT vs. PHDBT	NODAT vs. ND
Cardiovascular, *n* (%)	14 (17)	38 (17)	10 (4)	1.00	**<0.001**
Pulmonary, *n* (%)	17 (21)	45 (20)	29 (11)	1.00	**0.04**

Abbreviations: ND, non‐diabetes; NODAT, new‐onset diabetes after transplantation; PHDBT, prior history of diabetes before transplantation.

**TABLE 5:** Comparison of medication for diabetes after liver transplantation.

	NODAT	PHDBT	*p*
	*N* = 83	*N* = 225	
Patients who could stop the medication for diabetes, *n* (%)	43 (52)	15 (7)	**<0.001**

Abbreviations: NODAT, new‐onset diabetes after transplantation; PHDBT, prior history of diabetes before transplantation.

### 3.4 Prognostic factors in patients with NODAT

Among the patients with NODAT, rejection within 90 days after LT was a significantly risk factor for both graft loss (aHR 3.42, 95% CI 1.02–11.53, *p* = 0.04) and patient death (aHR 5.45, 95% CI 1.15–25.89, *p* = 0.03) after LT in the multivariable analysis (Table 6).

**TABLE 6:** Risks for 3‐year graft loss and patient death after liver transplantation in patients with NODAT.

Factors	aHR	95% CI	*p* Value
Graft loss			
Rejection within 90 days after LT	3.42	1.02–11.53	**0.04**
Duration of corticosteroids after LT	1.00	1.00–1.00	0.24
Patients who could stop corticosteroids	1.48	0.33–6.48	0.60
MELD score at LT	1.05	0.99–1.12	0.09
Recipient race Black [ref. White]	2.41	0.73–7.99	0.15
Patient death			
Rejection within 90 days after LT	5.45	1.15–25.89	**0.03**
Duration of corticosteroids after LT	1.00	1.00–1.00	0.06
Patients who could stop corticosteroids	0.96	0.22–4.32	0.96
MELD score at LT	1.06	0.99–1.13	0.11
Recipient race Black [ref. White]	2.14	0.59–7.77	0.24
HCC	1.87	0.55–6.37	0.31
ICU stay after LT	1.13	0.99–1.29	0.06

Abbreviations: aHR, adjusted hazard ratio; CI, confidence interval; HCC, hepatocellular carcinoma; ICU, intensive care unit; LT, liver transplant; MELD, model for end‐stage liver disease; NODAT, new‐onset diabetes after transplantation.

## 4 DISCUSSION

In our series, 15% of LT patients developed NODAT, who showed a higher proportion of cholestatic liver disease as their primary liver disease compared to those without diabetes. Multivariable analysis showed tacrolimus trough in the first week, higher HbA1c within normal limit, and early rejection after LT were associated with NODAT. After risk‐adjusted analysis, NODAT was a risk factor for graft loss and patient death similar to PHDBT. Of note, NODAT increased risks of post‐LT cardiovascular and/or pulmonary complications.

According to a large meta‐analysis by Chin et al., the incident rates of NODAT at 3, 6, and 12 months after LT were 15.5%, 16.1%, and 18.3%, respectively.^20^ It was consistent with our results. It was known that tacrolimus inhibits insulin‐mediated inactivation of hepatic glycogenolysis, causes a reduction in human pancreatic ductal cells, and inhibits glucose‐stimulated insulin secretion.^21^ Aravinthan et al. analyzed 2209 patients who received LT; they found that the use of tacrolimus was independently associated with NODAT development (OR 2.76).^22^ Recently, Ling et al. demonstrated that tacrolimus caused hepatic insulin resistance and triglyceride accumulation through insulin receptor substrate (IRS)2/AKT and sterol regulatory element binding protein (SREBP1) signaling, and respectively via CREB‐regulated transcription coactivator 2 (CRTC2) in mice.^23^ Meta‐analysis using 11 randomized controlled trials showed tacrolimus to be superior to ciclosporin in terms of patient mortality and hypertension, while ciclosporin was superior in terms of NODAT.^24^ In this study, five patients who received ciclosporin as an initial immunosuppression were excluded from this study, because the limited number of patients with ciclosporin did not allow comparisons with those with tacrolimus.

Instead, we focused on the possible effects of initial tacrolimus trough levels on the incidence of NODAT. Our study showed that tacrolimus trough in the first week was an independent risk factor for NODAT, which concurred with the findings from other studies. Song et al. calculated the mean trough concentration of tacrolimus in the year of diabetes diagnosis patients with NODAT or in the last year of the follow‐up in patients without NODAT.^25^ They reported that the mean tacrolimus of patients with NODAT was significantly higher than that of patients without NODAT and maintaining a tacrolimus value below 5.89 ng/mL after LT decreased risks for NODAT.^25^ Yagi et al. reported that a tacrolimus trough level ≥8 ng/mL 3 months after LDLT was an independent risk factor for NODAT in the multivariable analysis.^26^

Another important finding of this study was that an early rejection after LT was an independent risk factor for NODAT, which was consistent with the previous study.^9^ Patients with NODAT had a higher proportion of patients who received corticosteroids for a longer time. This might be related to a higher proportion of patients with cholestatic disease as their primary liver disease in the NODAT group. Corticosteroids have diabetogenic effects, which are insulin resistance and increased hepatic gluconeogenesis.^27^ Previous reports showed that withdrawal of glucocorticoids after LT might reduce the risk of NODAT.^16^, ^27^ It was reported that basiliximab might decrease risks for NODAT due to a decrease of steroids or a dose of tacrolimus.^16^, ^17^

Also, higher HbA1c even within normal limit was an independent risk factor for NODAT, and patients with NODAT showed a higher proportion of severe encephalopathy in univariable analysis although not significant in multivariable analysis compared to patients with NODAT to those without diabetes. Their higher HbA1c might be associated with glucose intolerance before LT. According to the study of 2248 patients who had received LT without pretransplant diabetes based on the National Health Insurance Research Database of Taiwan, encephalopathy was an important preoperative risk factor for NODAT (aHR, 1.54).^28^ Regarding other risk factors for NODAT, several previous studies have reported male,^29^ older age,^16^, ^26^, ^30^ family history,^29^ HCV infection,^29^ NASH,^22^, ^30^ high BMI (obesity),^16^, ^29^ and graft volume,^16^ which were controversial and not consistent in those reports. In our study, BMI, older age, and NASH were significant factors associated with PHDBT but not with NODAT.

We also found that NODAT was a risk factor for post‐LT mortality similar to PHDBT and increased risks of post‐LT cardiovascular and/or pulmonary complications. LV et al. reported that patients with NODAT showed higher mortality (mortality rate 40% vs. 22%) and an increased incidence of bacterial infection, and chronic renal insufficiency compared to patients without NODAT.^17^ They also demonstrated that the proportion of lung infection and multiple organ failure were more frequent in the NODAT group as a cause of death after LT,^17^ which was consistent with our study. Conversely, other studies reported that NODAT was neither associated with post‐LT mortality^15^, ^16^ nor post‐LT complications.^15^ According to the study by Moon et al., they classified 778 LT patients into four groups: patients with pre‐LT diabetes (20.4%), sustained NODAT (sustained 6 months or more after LT) (36.5%), transitory NODAT (temporarily existed 1 to 6 months after LT) (13.9%), and normal (29.2%). They concluded that the sustained NODAT was associated with a significantly higher rate of death due to infection as well as graft failure due to chronic rejection and late‐onset hepatic artery thrombosis.^31^ Another study about 994 LT patients, which had distinguished sustained NODAT from transient NODAT, showed that sustained NODAT is associated with long‐term major cardiovascular events.^30^ Although NODAT was not distinguished sustained from transient in our study, these classifications or definitions might be the cause of different results in each report.

Compared to patients who had diabetes before LT, interestingly, patients with NODAT showed a significantly higher proportion of patients who could discontinue diabetic therapies. DiRECT trial, which is RCT in the United Kingdom, showed that weight loss could achieve remission of type 2 diabetes, but this efficacy was less likely with longer durations of contracting diabetes.^32^ Our study showed that patients with NODAT were more likely to discontinue diabetic therapies. Future studies would be warranted to elucidate the reversibility of NODAT.

Regarding the poor prognostic factor among the patients who developed NODAT, rejection within 90 days after LT was a significant risk factor for both graft loss and patient death. In all cohorts including PHDBT and ND, interestingly, rejection within 90 days after LT was not a significant prognostic factor in this study. The patients with NODAT who have experienced rejection within 90 days after LT might need close follow‐up after LT.

There are several limitations in our study. This is a retrospective, single‐center analysis with a small sample size. Consequently, we could not adjust patient backgrounds among groups and could not evaluate the dose effects of corticosteroids after LT because the dose of corticosteroids might fluctuate during post‐LT course due to increase or decrease, and steroid pulse treatment. Also, the duration of NODAT was not evaluated. Despite these limitations, this study provides important insights into the risk stratification for NODAT and its impact on post‐LT outcomes.

In conclusion, NODAT was associated with worse post‐transplant outcomes. Since high initial tacrolimus concentration and episodes of early rejection were considered as risk factors for NODAT, careful immunosuppression management would be important to decrease its risk. Recently, LT patient populations have become older and the number of patients with NASH has been increasing. Because risks of cardiopulmonary complications were higher in patients with NODAT, pretransplant assessments and risk stratifications for possible underlying modalities in those patients would be crucial. Further investigations regarding the long‐term disease course of NODAT would be warranted to better understand its prognosis in LT patients.

## AUTHOR CONTRIBUTIONS

Shingo Shimada and Shunji Nagai contributed to the study concept/design, and drafting of this article. Shingo Shimada and Katsunori Miyake contributed to data collection/acquisition. Shingo Shimada and Shunji Nagai contributed to data analysis/interpretation. Deepak Venkat, Humberto Gonzalez, Dilip Moonka, Atsushi Yoshida, and Marwan Abouljoud contributed to the drafting and critical revision of this article. All authors have approved the final article.

## FUNDING INFORMATION

Nothing to disclose. This work received no financial support.

## CONFLICT OF INTEREST STATEMENT

The authors of this manuscript have no conflicts of interest to disclose.

## ETHICS STATEMENT

Approval of the research protocol: This study was conducted in accordance with the ethical principles of the Declaration of Helsinki and local laws and regulations. Study protocols were approved by the HFHS Institutional Review Board (#15051).

Informed Consent: Requirements for written informed consent were waived due to the de‐identified and observational nature of the data.

Registry and the Registration No. of the study/trial: N/A.

Animal Studies: N/A.

## Supporting information


Table S1.

## DISCUSSANT

### Professor Tomoharu Yoshizumi

Congratulations for your excellent presentation. We realized that we should treat NODAT properly after liver transplant because you showed that the NODAT was a risk factor of graft loss and of patient death after liver transplant. I have a couple of questions.

NODAT patient had a higher value of tacrolimus trough level, but also a higher incidence of acute cellular rejection. It seems curious for me. Do you know what's the reason of this finding?

The second question is, in 30% of NODAT group, steroids are not stopped, but the treatment for NODAT is to stop the steroid. What is the reason to continue the steroid? Is it to prevent a primary disease like PBC or PSC?

### Dr. Shingo Shimada

Thank you for a very important question. Rejection might be associated with multiple other factors such as patient age, primary disease, mycophenolate use, and induction immunosuppression therapies. Basically, mycophenolate was used in the immunosuppression regimen, but a small number of patients showed withdrawal of mycophenolate at discharge. Since the dosage might fluctuate during the course of each case, it was not included in this study. Our study found high tacrolimus blood levels and a high incidence of ACR in the NODAT group. However, the patient with higher blood levels of tacrolimus did not necessarily show ACR. We think that high tacrolimus blood level and high incidence of ACR were independent events, respectively.

Of the 24 cases of continued steroid use in NODAT, seven were used to prevent the recurrence of the primary disease. Among the remaining 17 cases, 11 cases, which is 65% of the patients, showed biopsy‐proven rejection during the course. We are also aware that steroid dosage should affect NODAT. We can evaluate the base daily dosage of steroids. However, in some cases, additional dosage is given due to rejection or other reasons. It was difficult to evaluate the total dosage of steroids objectively.

### Professor Takeaki Ishizawa

I would like to congratulate Dr. Shimada for tackling this important issue in the current situation of liver transplantation, where lifetime of post‐transplant patients is getting longer and longer.

Regarding your presentation, I am a little concerned about the overlaps between the development of early acute rejection and postoperative diabetes because the early acute rejection is known as an independent risk factor. Rejection followed by the strong anti‐immunotherapy is a possible risk factor for development of diabetes and also poor prognosis. Is it possible to show us any findings which could suggest a direct association of NODAT with a poor postoperative graft or patient survival, for example, the pathological proof of the fat infiltration on the transplanted liver or something? Or was the other factor [something] like severe infection rather than liver function associated with the poor postoperative survival?

### Dr. Shingo Shimada

I think it's very important, but it might be difficult. In this study, rejection was not a significant covariate for graft and patient survival in univariable analysis. Although the rejection at 30 days and at 90 days after liver transplant were only evaluated in this study, we did not include rejection in multivariable analysis. Thus, we confirmed by bivariable analysis, including NODAT and rejection.

NODAT was associated with worse graft and patient survival compared to non‐diabetes, which means the risk was adjusted for acute rejection, and NODAT remained as an independent factor. However, any specific pathological findings were not evaluated in this study. Instead, we checked that the cause of patient death showed a higher incidence of infection in NODAT patients.

### Dr. Yukiko Kosai‐Fujimoto

I want to ask you about the screening for the NODAT. In your slides, you showed that there are some diagnosis criteria and there are things like blood sugar and HbA1c. When do you measure those factors in your outpatient clinic? Because especially in the early stage, it seems there are more hurdles to check all the factors in every visit of the patients.

### Dr. Shingo Shimada

Most patients come to our hospital after discharge, at 1 month, 3 months, and 6 months after liver transplant. During visits to our hospital, the family medicine or hepatologist does a close follow‐up. So, we can check those factors at the outpatient clinic.
