# Supplementary material for: Clinical characteristics of new‐onset diabetes after liver transplantation and outcomes
Source: Ann Gastroenterol Surg. 2024 Jan 19;8(3):383–93. doi: 10.1002/ags3.12775 (PMC11066488; doi:10.1002/ags3.12775)
Supplement: Supplementary file 1 — Table S1. [file AGS3-8-383-s001.docx]

| **Table S1. Cause of 3-year mortality in patients who underwent LT** | | | | |
| --- | --- | --- | --- | --- |
|  | NODAT | PHDBT | ND |  |
| n | n=14 | n=25 | n=11 |  |
| Liver failure, n (%) | 1 (7) | 3 (12) | 1 (9) |  |
| Infection, n (%) | 5 (36) | 5 (20) | 2 (18) |  |
| Malignancy, n (%) | 4 (29) | 4 (16) | 5 (45) |  |
| GVHD, n (%) | 1 (7) | 2 (8) | 0 (0) |  |
| Renal failure, n (%) | 1 (7) | 2 (8) | 0 (0) |  |
| Others, n (%) | 2 (14) | 9 (36) | 3 (27) |  |

Abbreviations: GVHD; graft versus host disease, LT; liver transplantation.
